# A study of fracture lines distribution characteristics in complete articular fractures of the patella

**DOI:** 10.3389/fsurg.2023.1284479

**Published:** 2023-10-31

**Authors:** Xiong Wang, Shuming Zi, Wenqiang Wei, Qiang Yao, Liehu Cao

**Affiliations:** Department of Orthopedics Trauma, Shanghai Baoshan Luodian Hospital, Shanghai, China

**Keywords:** patella fracture, articular, morphology, fracture line, map

## Abstract

**Objective:**

The objective of this study was to unveil the characteristics of fracture lines distribution and explore its clinical significance of complete articular fractures of the patella.

**Methods:**

A consecutive series of image data from 88 patients with complete articular patella fractures were retrospectively included. Three-dimensional reconstruction images of the patella fractures were created and collected. Subsequently, these reconstructed images were visually overlaid onto a standard anterior and posterior patella template. The fracture lines were then identified, traced onto the template, and utilized to generate patella fracture maps. Furthermore, the incidence rate of patella fracture lines involving the distal pole was analyzed.

**Results:**

The maps depict the fracture lines of complete articular patella fractures. For simple and complex patella fractures, the primary fracture lines primarily converge within the Middle and Lower regions, exhibiting a transverse pattern. Conversely, the primary fracture lines in comminuted patella fractures are randomly dispersed across the patella. Examining the maps, approximately 63.6% (56/88) of complete articular patella fractures exhibited involvement of the distal pole in the anterior view, while 48.9% (43/88) displayed distal pole fractures in the posterior view. The incidence of distal pole injury increased progressively with the severity of patella fractures.

**Conclusion:**

The patterns and distribution of fracture lines in cases of complete articular patella fractures are prominently illustrated on the constructed fracture maps. Familiarity with these common characteristics of complete articular patella fracture, especially with the distal pole injury, can aid surgeons in developing preoperative planning, executing surgical strategies effectively, and reducing inappropriate treatment.

## Background

The patella, being the largest sesamoid bone in the human body, plays a crucial role in stabilizing knee joint function, supporting walking, and augmenting quadriceps strength (1). Surgical intervention is necessary when patella fracture hampers knee function (2). Patella fractures generally occur in patients over the age of 50 and account for approximately 1% of all fractures (2, 3). These fractures are commonly caused by direct or indirect trauma, with a significant proportion involving the articular surface. Direct impact or falls often result in comminuted patella fractures, while indirect injuries such as excessive quadriceps contraction lead to transverse fractures. Fractures involving the articular surface, comminution, or displacement greater than 1–4 mm are indications for surgical intervention (4, 5). Various techniques including Kirschner wires, tension band fixation, cannulated screws, plates, and suture anchors have been widely employed for treating patella fractures (6–8). However, postoperative complications such as skin irritation, chronic pain, internal fixation failure, traumatic osteoarthritis, and the need for secondary operations are relatively common due to a lack of comprehensive understanding of patella fractures (9, 10). Therefore, it is essential to have a comprehensive understanding of the characteristics of patella fractures in order to select appropriate management strategies and achieve favorable outcomes.

While conventional x-ray imaging serves as the standard diagnostic method for patella fractures, the position of the patient's knee and the projection angle may affect the accuracy of fracture characterization (11). Computed tomography (CT) scans have become frequently utilized in orthopedics due to their ability to provide fast and precise examination details of fractures. Three-dimensional (3D) CT reconstruction aids physicians in identifying the location of fracture lines and the morphology of different fragments (12). Previous studies have demonstrated that inaccurate evaluation of fracture patterns lead to inappropriate treatment and subsequent functional impairments of the patella (13). Researches have shown that fracture line maps can assist surgeons in understanding fracture morphology, facilitating preoperative planning, and determining suitable treatment methods (14–16).

In this study, we employed CT mapping techniques to establish frequency distribution maps of patella fracture lines in anterior and posterior views. The objective of this research was to further characterize the common distribution characteristics of patella fracture lines and provide valuable references for surgeons during preoperative planning and select appropriate treatment options.

## Clinical data

### General information

**Figure 1:**
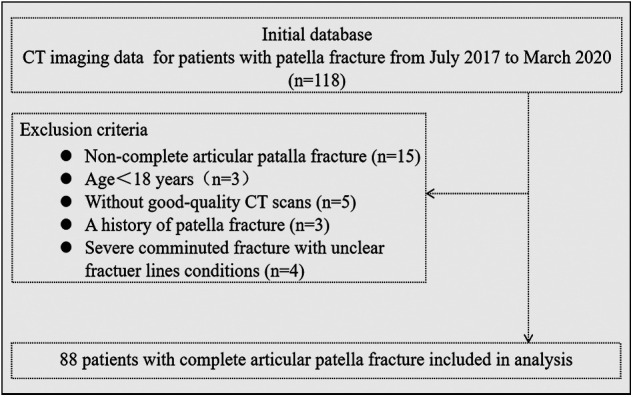
Flow diagram of case in- and exclusion.

From July 2017 to March 2020, CT imaging data from 118 patients with patella fractures were collected. Experienced orthopedic physicians searched the Picture Archiving and Communication System (PACS) database to obtain the data. The inclusion criteria for this study were as follows: patients with complete articular patella fractures (OTA/AO 34C type), aged 18 years or older, and high-quality CT scans (slice thickness between 0.5 mm and 1.0 mm). Patients with pathological fractures, a history of previous patella fractures, or severe comminuted fractures with unclear fracture lines were excluded. Two experienced orthopedic surgeons evaluated and collected all the CT data of the patients. Ultimately, 88 cases of complete articular patella fractures were included in the study (Figure 1). Among these cases, there were 40 males and 48 females, with a mean age of 57.2 years (ranging from 25 to 82 years). The left side was affected in 47 patients, while the right side was affected in 41 patients. The fractures resulted from falls from heights in 57 patients, vehicle accidents in 26 patients, and other causes in 5 patients. The study received approval from the institutional review board of the hospital, and informed consent was obtained from all participants.

### Fracture mapping

**Figure 2:**
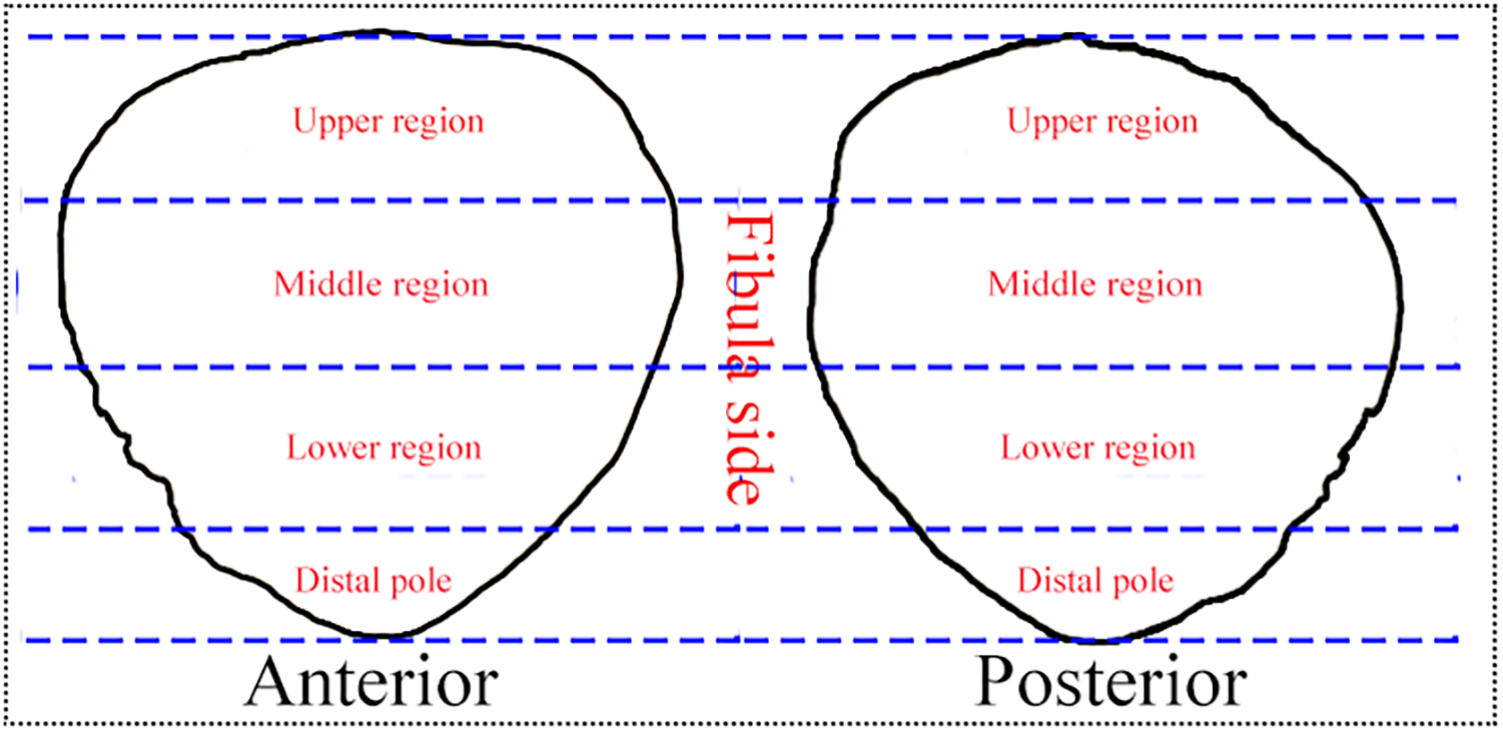
The four regions of standard patella template.

The Digital Imaging and Communications in Medicine (DICOM) format data of a healthy young man's left knee joint was imported into the E-3D Digital Orthopaedics software (developed by Central South University, Changsha, China). Using this software, image and standard modeling were performed after 3D reconstruction. The tibia, femur, and fibula were removed, leaving only the patella. The “standard anterior and posterior” views of the patella were selected within the software and utilized as the template for analysis (12). In order to facilitate a more detailed exploration of the distribution regions of patella fracture lines, the patella template was divided into four regions: upper region, middle region, lower region, and distal pole (Figure 2).

**Figure 3:**
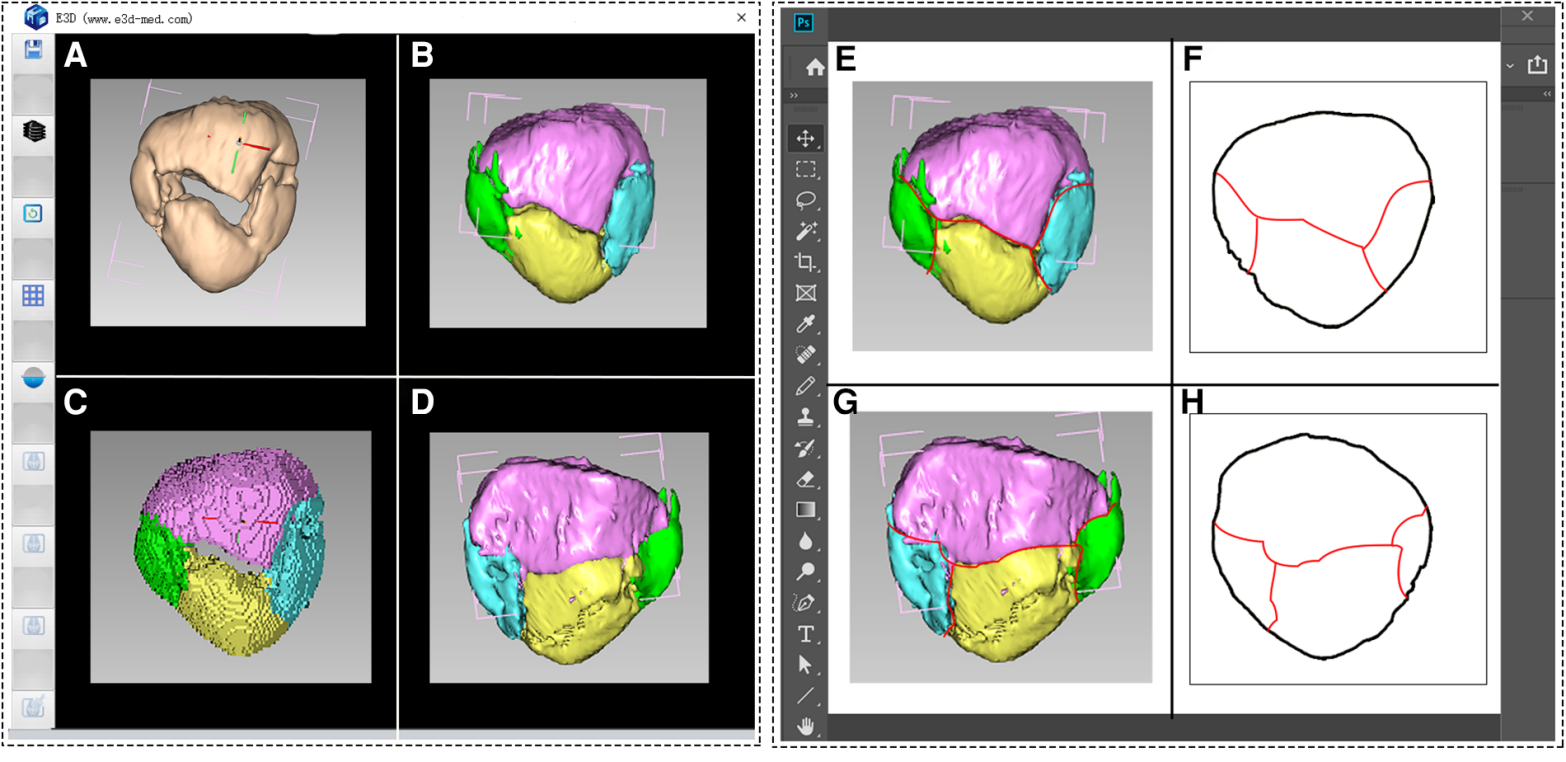
The process of virtual reduction of patella fracture in E-3D software, and drawing and superimposing fracture lines to template in Photoshop software. (A–D) 3D reconstruction and virtual reduction of patella fracture. (E–H) Drawing fracture lines and superimposing them to the anterior and posterior templates.

Subsequently, the DICOM format data of the 88 included cases were individually loaded into the E-3D software. Through this software, 3D reconstruction and virtual reduction of patella fractures were conducted, matching them with the standard template to represent the details of the fracture lines. For right-sided patella fractures, virtual reduction was performed, followed by a horizontal flip to match the left-sided model. The resulting 3D reconstruction images were then imported into Adobe Photoshop CC 2019 software (Adobe Systems Incorporated, San Jose, CA, USA). Using the pencil tool in Photoshop, individual fracture lines were meticulously drawn on both the anterior and posterior views (Figure 3).

**Figure 4:**
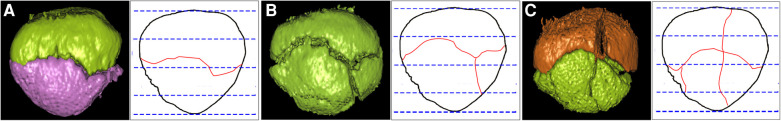
The definition of three Groups based on the fracture lines type. (**A**) Group A refers to a simple patella fracture, with one primary fracture line. (**B**) Group B refers to a complex patella fracture, with one primary fracture line and some secondary fracture lines. (**C**) Group C refers to a comminuted patella fracture, with more than two primary fracture lines.

By overlaying all the individual fracture lines onto the standard patella template, a comprehensive map of the fracture lines was obtained. To examine the features of fracture line distribution regions and fracture patterns, three groups were defined based on the type of fracture lines. Group A: This group consisted of cases with simple patella fractures characterized by one primary fracture line. Group B: Cases with complex patella fractures were included in this group, featuring one primary fracture line along with multiple secondary fracture lines. Group C: Comminuted patella fractures, characterized by more than two primary fracture lines (Figure 4). These groupings allowed for a detailed analysis of the fracture line distribution regions and patterns observed in the complete articular patella fractures.

### Statistical analysis

Continuous variables such as patient characteristics are presented as mean ± standard deviation. Categorical variables are presented as frequencies or proportions. Descriptive analyses were used to construct the characteristics of fracture lines distribution of complete articular patella fractures.

## Results

**Figure 5:**
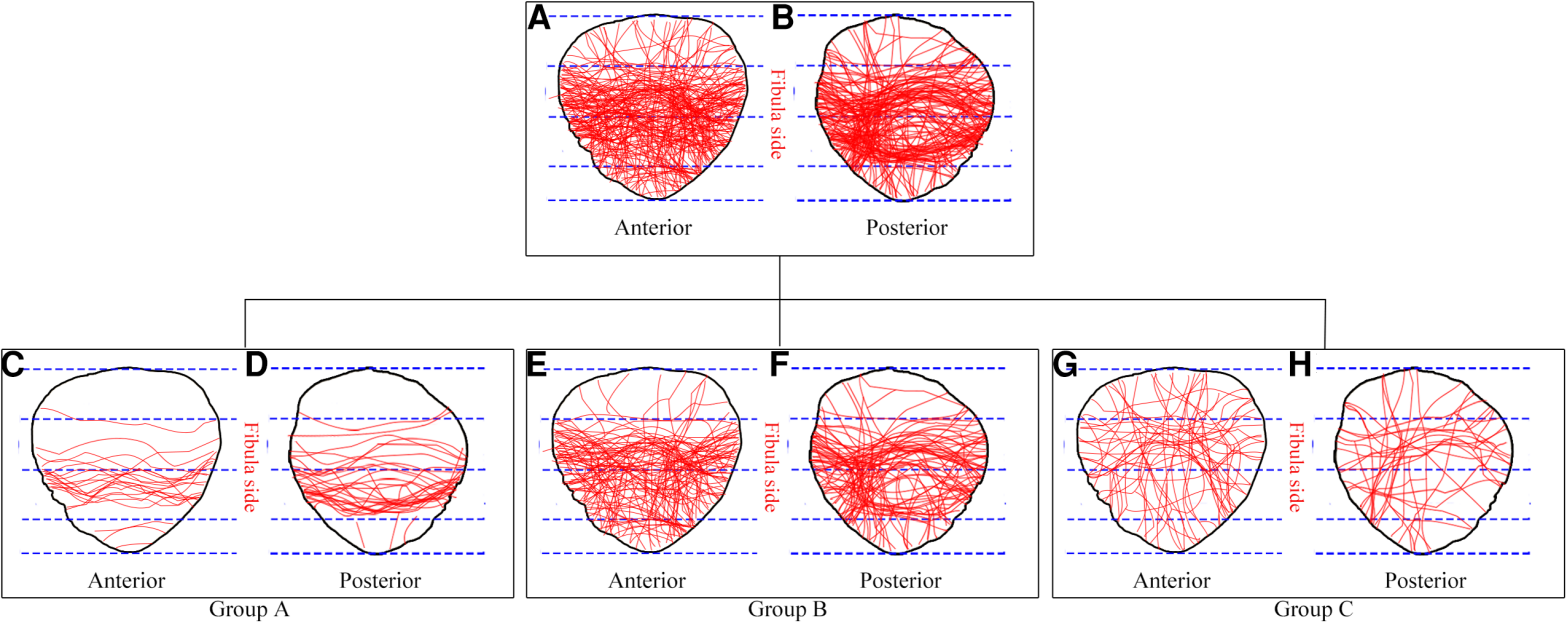
The distribution characteristics of complete articular patella fractures lines in the anterior and posterior views. (**A,B**) Maps of all fracture lines together. (**C,D**) Maps of fracture lines distribution in group A. (**E,F**) Maps of fracture lines distribution in group B. (**G,H**) Maps of fracture lines distribution in group C.

The results of this study include the fracture line maps of the 88 cases with complete articular patella fractures, presented on both the anterior and posterior patella templates (Figure 5). Viewing the distribution characteristics of these maps, we found that the fracture line distribution characteristics and regions were not identical on the anterior and posterior sides. On the anterior side, 19 cases showed as Group A, including 3 cases with distal pole injury. 51 cases showed as Group B, including 37 cases with distal pole injury. 18 cases showed as Group C, including 16 cases with distal pole injury. On the posterior side, 28 cases showed as Group A, including 3 cases with distal pole injury. 47 cases showed as Group B, including 28 cases with distal pole injury. 13 cases showed as Group C, including 12 cases with distal pole injury.

The primary fracture lines distribution regions were summarized in Table 1. For simple patella fractures (Group A), the primary fracture lines were primarily concentrated between the middle and lower regions in both the anterior and posterior views. This distribution pattern indicates a transverse fracture pattern. In complex patella fractures (Group B), the primary fracture lines were predominantly located between the middle and lower regions, exhibiting a transverse fracture pattern similar to Group A. However, in addition to the primary fracture line, there were also multiple secondary fracture lines involved in the lower and distal pole regions. Comminuted patella fractures (Group C) displayed a random dispersion of primary fracture lines across the patella in both the anterior and posterior views.

**Table 1:** The features of primary fracture lines location in the anterior and posterior views of the patella.

Region	Anterior view of patella			Posterior view of patella		
	Group A (*n* = 19)	Group B (*n* = 51)	Group C (*n* = 18)	Group A (*n* = 28)	Group B (*n* = 47)	Group C (*n* = 13)
Three regions	/	/	18	/	/	13
Upper	0	0	/	0	0	/
U&M	1	2	/	2	3	/
Middle	1	16	/	2	15	/
M&L	7	28	/	7	26	/
Lower	10	5	/	17	3	/
Distal pole involved	3 (15.7%)	37 (72.5%)	16 (88.9%)	3 (10.7%)	28 (59.6%)	12 (92.3%)

U&M, the junction of the upper and middle regions; M&L, the junction of the middle and lower regions.

Of note, about 63.6% (56/88) complete articular patella fractures associated with the distal pole fracture in the anterior view, whereas 48.9% (43/88) fractures associated with the distal pole fracture in the posterior view, which was easy to neglect in traditional x-ray examination. It showed the inconsistency of fracture lines between observations from the anterior and posterior views. Additionally, the incidence rate of involved distal pole injury was gradually increasing with the severity of patella fractures (Table 1, Group C > Group B > Group A).

## Discussion

At present, the OTA/AO classification system, based on fracture line orientation and complexity of the fracture with plain radiography, is the most popular and accepted fracture classification system (17). However, as a result of the superimposition of the patella fragments and femoral condyle, it is hard to accurately identify the significant morphologic details and fracture lines orientation. With the development of CT scan and 3D reconstruction techniques, a complete explanation of the fracture patterns and characteristics can be achieved (12, 18). Cole et al. conducted the fracture mapping technology by superimposing fracture lines from axial CT scan to reveal the fracture lines conditions and develop preoperative planning (14). However, the maps mentioned above could not reflect the whole fracture characteristics of the intact bone structure.

In the present study, 3D construction and virtual reduction of patella fracture were performed, and the reconstruction images were utilized to create fracture maps. The patella fracture maps demonstrated that the transverse fracture was the most common fracture type, which was similar to those of previously published results of Misir et al. (19). The primary fracture lines of Group A and B were mostly concentrated between the region M and L. Whereas the primary fracture lines of Group C were randomly spread. Researches have found that the fracture maps could assist surgeons to make preoperative planning and choose appropriate surgical strategies (15, 16). The tension band wiring with kirschner wire or cannulated screw was suitable for simple transverse fracture pattern (Group A). It can transform tensile forces into compressive forces during knee flexion and provide stable fixation (20–22). However, it may cause internal fixation failure in complex patella fracture like Group B. Because the second fracture lines of patella would lead to fragment displacement and fixation stability lost. Previous studies have proved that tension band wiring combined with the wire loop cerclage or screws fixation could provide stable fixation for complex patella fracture (13, 23). However, the surgical methods mentioned above may be hardly to fix the comminuted patella fractures like Group C, because the comminuted fracture fragments are prone to displacement and lose fixation (2, 24). Taylor et al. reported that after accurate evaluation of fracture patterns, fixed-angle plates and mesh plates were successfully utilized in a severe complex comminuted patella fracture, all of which achieved superior function outcomes compared with traditional tension band wiring (7). Ellwein et al. discovered that the patella locking plating was a safe and effective treatment for comminuted patella fracture, and most patients achieved better range of motion (140°–143°), higher Kujala score (97 scores) (25). Chen et al. found that the application of the Nice knots as an auxiliary reduction technique in displaced comminuted patella fractures was associated with reduced surgical time, decreased intraoperative blood loss and satisfactory postoperative outcome (26). Another study discovered that anatomically contoured locking plates allow secure fixation of the comminuted fracture fragments compared with tension band wiring, it also could provide good functional results and low complication rates (27). Siljander et al. revealed that using the low-profile mesh plating technique, complicated patella fracture with the distal pole injury can achieve early rehabilitation, fewer complications, and better recovery (28).

In addition, through the fracture maps, we discovered that the distribution characteristics of fracture lines were inconsistent in the anterior and posterior views. Approximately 63.6% of the complete articular patella fracture were observed with distal pole fractures in the anterior view, about 48.9% in the posterior view. And the incidence rate of involved distal pole fracture was gradually increasing with the severity of patella fracture (Table 1). The distal pole of the patella is very important in maintaining the stability of the overall knee structure (1). The combined distal pole fractures were easily to overlooked in the traditional x-ray examination and seldom to manage in clinical practice, which may affect the function prognosis and lead to fixation failure. Non-rigid internal fixation of the inferior pole fracture would disrupt the extensor mechanism, which results in considerable functional disability (29). A research revealed that the anchor and Krackow-“8” Suture for the fixation of distal pole fracture was an effective operation method and better surgical outcome (30). Suture bridge anchor fixation was also successfully used in the comminuted inferior pole fracture patella, which showed good bony union and satisfactory clinical outcome (8). Therefore, clinical surgeons should pay enough attention before preoperative evaluation and planning for complete articular patella fracture, especially for those associated with distal pole injury, and reduce inappropriate treatment options.

This study had several limitations that should be noted. First, the sample size was small because many patients without CT scans were excluded, and this might have influenced the universality of the results. Second, the distribution characteristics of fracture lines with different genders and injury mechanisms were not evaluated. Third, the zone of we interest employed in the patella fractures could not match the template perfectly because of the anatomical variability nature of the patella. Consequently, the extracted fracture lines overlapped with the standard template, slightly losing the authenticity of the real fracture morphology. Therefore, a larger sample size and multi-factor study are needed for future investigations. Despite these limitations, we still think that a comprehensive understanding of patella fracture patterns could provide valuable information and clinical reference for surgeons during preoperative planning.

## Conclusion

This study describes fracture lines distribution characteristics of complete articular patella fractures. Knowledge of these can provide surgeons with a comprehensive understanding of fracture patterns and the features of distal pole injury. It can assist surgeons in developing preoperative planning, selecting optimal surgical strategies, and reducing inappropriate treatment.

## Data Availability

The original contributions presented in the study are included in the article/Supplementary Material, further inquiries can be directed to the corresponding author.
